# Differential Detection of *Echinococcus* Spp. Copro-DNA by Nested-PCR in Domestic and Wild Definitive Hosts in Moghan Plain, Iran

**Published:** 2013

**Authors:** I Mobedi, M Zare-Bidaki, MR Siavashi, SR Naddaf, EB Kia, M Mahmoudi

**Affiliations:** 1 Dept. of Medical Parasitology and Mycology, School of Public Health, Tehran University of Medical Sciences, Tehran, Iran; 2 Immunology of Infectious Diseases Research Center (IIDRC), Rafsanjan University of Medical Sciences, Rafsanjan, Iran; 3 Department of Medical Parasitology, Pasteur Institute of Iran, Tehran, Iran; 4 Dept. of Epidemiology and Biostatistics, School of Public Health, Tehran University of Medical Sciences, Tehran, Iran

**Keywords:** *Echinococcus*, Copro-DNA, Canids, Iran

## Abstract

**Background:**

Despite *Echinococcus granulosus*, there are merely two old reports of *E. multilocularis* infection among Iranian canids of Moghan Plain, the only area known endemic for the species. We detected specific DNA markers in fecal samples by PCR (Copro-PCR) for differential diagnosis of *Echinococcus* species in living canids.

**Methods:**

Totally 144 fecal samples from domestic dogs, red foxes and a golden jackal were examined for genus-specific *Echinococcus* coproantigens using ELISA. Forty two positive or ambiguous samples were further examined for *Echinococcus* species-specific DNA markers by two different set of nested-PCR.

**Results:**

Twenty five out of 144 (17.4%) animals were contaminated with *E. granulosus* including 14 (23.7%) domestic dogs, 10 (11.9%) red foxes and one (100%) golden jackal. But none of them harboured *E. multilocularis* species-specific Copro-DNA. The overall prevalence of *E. granulosus* and *E. multilocularis* infections in canids of the area was estimated to be 17.4% and 0.0%, respectively. There was a significant relation between the results of Copro-PCR and CA-ELISA.

**Conclusion:**

The lack of *E. multilocularis* infection, compared to previous reports may be due to the differences in used diagnostic methods and/or recently limited territories of wild canids and altered their food resources in this particular area.

## Introduction

Echinococcosis is a near-cosmopolitan zoonosis caused by adult or larval stages of tapeworms belonging to the genus *Echinococcus* (family Taeniidae; class Cestoda). Larval infection known as hydatid disease or hydatidosis is characterized by long term growth of hydatid cysts in the intermediate hosts including humans, herbivores and rodents .In the parasite life cycle, carnivores especially canids, serve as definitive hosts and harbor the adult worms in their intestines. The two major species of medical and public health importance are *Echinococcus granulosus* and *E. multilocularis*, which cause cystic echinococcosis and alveolar echinococcosis, respectively (1, 2).

*Echinococcus granulosus* infection in definitive hosts is common throughout Iran (3) and its adult worms have been detected in various carnivores from rural and urban areas of many Iranian provinces including Kerman, Khuzestan, Fars, Tehran, Kurdestan, Khorasan, Isfahan and several western provinces (3–6).

But in the case of *E. multilocularis* infection in Iran, there are merely two old reports indicating that canids might serve as definitive hosts in Moghan plain, the only area in the country known to be endemic for this species. The first report on canine infection to adult worms of *E. multilocularis*, dates back to 1971, in which 10% red foxes (3 0ut of 30) were found infected (7, 8). The next study on 130 wild and domestic carnivores in Ardebil province, northwestern Iran in 1992 revealed that 22.9% of red foxes and 16% of jackals and 50% wild cats (one out of 2) were infected with adult stages of *E. multilocularis* (9, 10). The diagnosis in both aforementioned studies was based on morphological characteristics of adult worms at necropsy.

Because of high similarity between eggs of *Echinococcus* and *Taenia* species, the differential diagnosis of *Echinococcus* infection in fecal samples of canids is impossible (11). Additionally, the characteristic small *Echinococcus* proglottids may be absent in the feces or be easily overlooked (2). By the end of the 1980's the only reliable technique for diagnosis of intestinal *Echinococcus* infection in definitive hosts was Intestinal Scraping Technique (IST) at necropsy and examination of scraped materials under stereoscope. IST with maximum sensitivity of 78% in optimal conditions (1) is based on investigation of the dead animal's intestine and visual identification of the adult worms, based on morphological features (11). IST is considered as an expensive, bio-hazardous and laborious diagnostic method and is not recommended for examination of domestic live animals. Moreover, since dogs and red foxes are known to be susceptible to *E. granulosus* and *E. multilocularis* species, they might simultaneously be infected with both species (1).

In recent years, two new techniques, based on detection of the parasitic copro-DNA molecules by PCR (Copro-PCR) and Copro-Antigens by Enzyme-Linked ImmunoSorbent Assays (CA-ELISA) in animal fecal samples were introduced for the diagnosis of *Echinococcus* infections in living carnivores (2).

Briefly, Copro-PCR tests, involve purification of eggs from fecal materials, extraction of DNA, and identification of species-specific target sequences using DNA primers. The target sequences are often directed towards ribosomal and mitochondrial DNA, as there is generally a higher level of genetic heterogeneity and mutation within these sequences, increasing the presence of species-specific determinants (12). The first PCR-based method for the detection of *E. multilocularis* DNA in fecal samples of foxes was developed by Bretagne et al. (13) and later modified and improved (11, 14–24). The method is now recommended as an alternative method to the routine IST. Detection of *E. multilocularis* Copro-DNA by nested-PCR has showed a specificity of 100% and average sensitivity of 89%, ranging from 78% to 100%, influenced by worm burden (11). In summary, although the wide use of PCR for field studies will largely depend on the facilities and the costs, however Copro-DNA detection is already accepted and used as a confirmation test for positive samples in CA-ELISA (16) or in selected cases, especially in living dogs and cats (2).

To date, most of studies on prevalence of intestinal helminth infections of carnivores in Iran were merely based on the traditional method of IST. But recently, Siavashi et al. (25) used CA-ELISA for detection of canine echinococcosis in three provinces of Iran and reported the specificity and sensitivity of the method to be 74% and 72%, respectively. In our similar study using the same method in Moghan Plain, 21.6% of canids were found to be infected with *Echinococcos* Spp. (6), however, CA-ELISA could not identify the tapeworm species.

This study was aimed to determine the prevalences of *E. granulosus* and *E. multilocularis* infections among canine definitive hosts using nested-PCR in Moghan Plain, northwestern Iran, the only area in the country labeled as endemic for *E. multilocularis* infection.

## Materials and Methods

### Study area

This study was performed in the Moghan Plain (local name: Dasht-e-Moghan) in the province of Ardebil, northwestern Iran, bordered in north and east by Azerbaijan Republic. The area is located between 39°0' and 39°36' north latitude and 46°52' and 48°21' east longitudes. The region covers 5245 Km^2^ and includes three counties namely Pars Abad, Bileh Savar, and Germi. The population is approximately 310,000 of urban, rural and nomadic people. Most of the people are from Azeri ethnic group and mainly practice agriculture and Stockbreeding. The Moghan Plain consists of the plains with altitudes as low as 32 meters and highlands with heights as high as 1023 meters above sea level and has an average annual precipitation of 222.76 millimeters.

### Samples

Totally 144 fecal samples including 59 from domestic dogs (*Canis lupus* f. *familiaris*) and 84 from red foxes (*Vulpes vulpes*), and one from a golden jackal (*Canis aureus*), were collected and examined for *Echinococcus* species by parasitological, serological and molecular methods.

The red foxes specimens were obtained either from rectums of necropsied foxes (n = 79) or from vicinity of red foxes dens (n = 5). Since fecal samples might contain infective eggs or proglottids of *E. granulosus* and *E. multilocularis*, all samples were frozen at least for one week in -70° C and then kept at -20° C until used.

The samples were first examined for genus-specific *Echinococcus* coproantigens using CA-ELISA as described before (6). Then 30 positive and 12 ambiguous samples (Table 1) were further investigated by two set of nested-PCR using specific primers.

**Table 1:** Distribution of samples according to host species and the results of CA-ELISA

Host species	Negative	Ambiguous	Positive	Total
Fox	67	4	13	84
Dog	34	9	16	59
Jackal	0	0	1	1
Total	101	13	30	144

### Copro-DNA nested PCR

#### a. DNA extraction from adult *Echinococcus* worms

DNA of an Iranian isolate of adult *Echinococcus* tapeworm was extracted and used as template to optimize the PCR, and as positive controls in PCR runs. For grinding of whole body of tapeworms in DNA extraction procedure, we evaluated five different methods including: a) using detergents; b) homogenizing by manual homogenizer; 3) sinking in liquid nitrogen followed by manual homogenizing; 4) squeezing adult worms between two glass slides; and 5) freezing in liquid nitrogen and thawing in hot water accompanied by manual homogenizing.

The best results were achieved in the last procedure. Then QIAamp DNA Mini Kit (Qiagen, Hilden, Germany) was used to extract genomic DNA from adult worms. Extracted DNA was solved in 100µL distilled water.

#### b. DNA extraction from fecal samples

Because of low concentration of Copro-DNA in extracts, all fecal samples were concentrated by a modified sedimentation technique prior to DNA extraction. Briefly, fecal specimens were suspended in normal saline, filtered through strainer and gauze, poured in 50-mL Falcon^®^ tubes and centrifuged one or more times in 1500 rpm for 10 minutes, until the supernatant came out clear.

DNA extraction from the sediments was performed using a commercial specific kit, QIAamp DNA Stool Mini Kit (Qiagen, Hilden, Germany) according to manufacturer protocols.

#### c. nested-PCR

According to recommendation of previous studies (2, 25), we merely used the PCR technique as a differential method and a confirmation test to CA-ELISA. Therefore, only positive or ambiguous (borderline) fecal samples in CA-ELISA (n = 42) were subjected to PCR to detect DNA molecules of *Echinococcus* species.

The extracted DNA samples were amplified with two nested-PCR protocols using the primers (as detailed in Table 2) and procedures described by Dinkel et al. (11) and Abbasi et al. (23) and optimized by some modifications in thermal cycles parameters and the concentrations of reagents as follows:

**Table 2:** The details of primers used in nested-PCR protocols

Species/target (Ref.)	Step	Name	Sequence	Product	Specificity
*E. multilocularis* /mitochondrial 12S rRNA gene (11)	1^st^	P60.for. p375.rev.	TTAAGATATATGTGGTACAGGATTAGATACCC AACCGAGGGTGACGGGCGGTGTGTACC	373 bp	Genus

	2^nd^	Pnest.for. Pnest.rev	CAATACCATATTACAACAATATTCCTATC ATATTTTGTAAGGTTGTTCTA	255 bp	Species
	
*E. granulosus* /EgG1 *Hae III* (24)	1^st^	Eg21: Eg22	ACACCACGCATGAGGATTAC ACCGAGCATTTGAAATGTTGC	269 bp	species

	2^nd^	Eg23 Eg24	GAATGCAAGCAGCAGATG GAGATGAGTGAGAAGGAGTG	133 bp	species

#### c.1- Nested-PCR for *E. multilocularis*

The PCR with both pairs of primers was carried out in a volume of 25 µL PCR reaction that contained 10 mM Tris-HCl, pH 8.3, 50 mM KCl, 1.5 mM MgCl_2,_ 200 M dNTP (each), 1U/reaction Taq DNA Polymerase, 5 l/reaction DNA Template and 20 pmol/reaction Primers (each).

The thermal profile optimized as 5 minutes at 95°C followed by 35 cycles, each of 45 seconds at 93°C, 35 seconds at 63.3°C, and one minute at 73°C and a final elongation phase for 5 minutes at 72°C.The

#### c.2- Nested-PCR for *E. granulosus*

In both two stages of PCR, reaction volumes of 25 µL contained 10 mM Tris-HCl, pH 8.3, 50 mM KCl, 1 mM MgCl_2,_ 200 M dNTP (each), 1U/reaction Taq DNA Polymerase, 5 l/reaction DNA Template and 20 pmol/reaction Primers (each).

The thermal cycles for both steps was as 2 minutes at 95°C followed by 35 cycles, each of 30 seconds at 95°C, 45 seconds at 55°C, and 45 seconds at 72°C and a final elongation phase for 7 minutes at 72°C. All PCR runs were performed using Mastercyclers^®^ thermocycler (Gradient 5331, version 2.03.31-09; Eppendorf AG, 22331 Hamburg). PCR products were subjected to electrophoresis on a 1% agarose gel in TAE buffer.

## Results

**Fig. 1:**
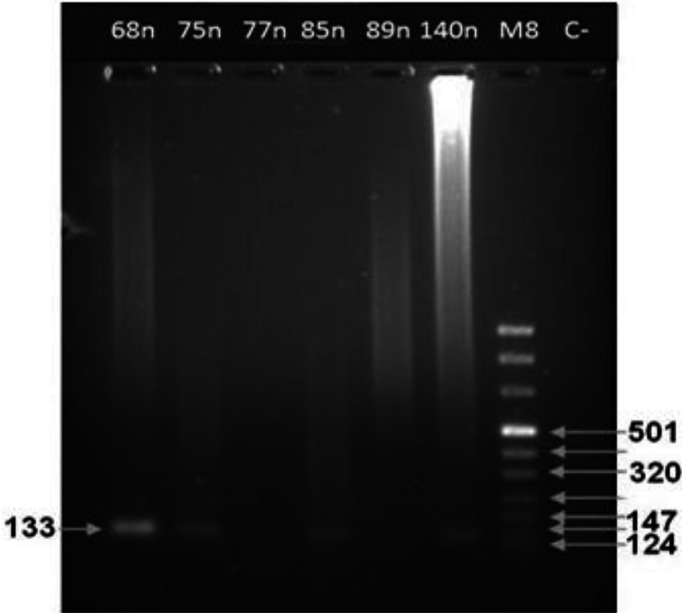
Representative results of second stage of nested-PCR with internal specific primers of Eg23 and Eg24 (see Table 2) on some fecal samples. Bands of a 133 base pair DNA product of *E. granulosus* were amplified in samples 68n, 75n, 85n and 140n, but not in samples 77n and 89n. Lane M8, DNA size marker; lane c-, negative control

Out of 42 samples subjected to nested-PCR, *E. granolosus* Copro-DNA was detected in 25 samples (Fig. 1). The 255-basepair amplicon of internal species-specific primers (Pnest. for. and Pnest.rew.) representative of *E. multilocularis* Copro-DNA, was not amplified in any sample. Although, the nonspecific products of *E. multilocularis* external primers (P60.for. and p375.rev.) were seen in four samples, but because of probable its amplification by *Mesocestoides* spp, *E. granulosus*, and or many other taenids (11) and of having been positive in early CA-ELISA, they could be evidence of *E. granulosus* infection.

Generally, according to the results of CA-ELISA (6) and Copro-PCR methods, we found that 25 out of 144 (17.4%) animals were contaminated with *E. granulosus* including 14 domestic dogs, 10 red foxes and one golden jackal.

There was a significant relation between the results of Copro-PCR and the net optical density (OD) values of samples run on a spectrophotometer at 450 nm wavelength in CA-ELISA (*P* = 0.041).

There was no significant relation between Copro-PCR results and the species of animal hosts, the geographical location of, and the seasons of sampling, as well as many other parasitic infections. But positive Copro-PCR results was statistically higher in animals with *Toxocara canis* (*P*< 0.05) and *Mesocestoides* spp. (*P*=0.075) infections.

Additionally, in preliminary parasitological examinations of fecal samples using wet mount, formol-ether and sucrose floatation methods, Taenia-like eggs were found in 10 (11.2%) dogs and 2 (3.4%) red foxes. The detailed parasitological results were presented in our other paper (26).

## Discussion

CA-ELISA is based on polyclonal *Echinococcus* genus-specific antibodies and is not able to differentiate *E. multilocularis* from *E. granulosus* in fecal samples (2). Therefore we used Copro-PCR to detect species-specific Copro-DNA and to determine the prevalences of *E. garnulosus* and *E. multilocularis* infections in canids of the study area.

As expected, Copro-PCR had high consistency with CA-ELISA results. There was a significant relation between Copro-PCR results and the net optical density (OD) values of specimens in CA-ELISA. Furthermore, positive Copro-PCR cases were higher, albeit statistically insignificant (*P*-value = 0.136), in positive CA-ELISA specimens than negative ones (66.7% vs. 41.7%).

Regarding the relation between the results of Copro-PCR and other parasitic infections, it is worth mentioning that among 12 samples harboring *Taenia* eggs, *Echinococcus* Copro-DNA was detected only in one dog fecal sample, indicating the high specificity of Copro-PCR method.

The statistically significant relation between the results of Copro-PCR with *Toxocara canis* and *Mesocestoides* spp. infections was probably due to their high prevalence rates of 34.9% and 48.3%, respectively, obtained in our parasitological investigations (26), as well as it may be an indicative of a strong co-infection phenomenon.

If the Copro-PCR that has higher sensitivity and specificity than CA-ELISA (25), is considered as gold standard test, the sensitivity of CA-ELISA method would be 80%. In this study it was impossible to estimate the specificity of CA-ELISA, as only positive and ambiguous specimens in CA-ELISA were subjected to Copro-PCR.

## Conclusion

The prevalence of *E. granulosus* in dogs and red foxes of the Moghan plain were 23.7%, 11.9%, respectively. The overall prevalences of *E. granulosus* and *E. multilocularis* infections in canids of the area is estimated to be 17.4% and 0.0%, respectively.

The lack of *E. multilocularis* infection in this study, compared to two previous works in 1971 and 1993 might be due to the differences in used diagnostic methods and/or extensive ecologic changes in recent years including the population growth and immigration, the establishment of new villages and towns around local rivers (Aras, Darehroud, Balharoud and aghbeiglar) and the building of new dams (Aras, Aslandouz and Khoda-afarin), new water reservoirs and irrigation networks. It seems that these factors could limited the territories of wild carnivores such as red foxes, jackals and wolves and also altered the their food resources as shifting from *Microtus* voles to other rodents e.g. *Meriones* spp, insects, birds and lizards in this particular area.
